# Exquisitely Constructing a Robust MOF with Dual Pore Sizes for Efficient CO_2_ Capture

**DOI:** 10.3390/molecules28176276

**Published:** 2023-08-28

**Authors:** Yanxi Li, Yuhua Bai, Zhuozheng Wang, Qihan Gong, Mengchen Li, Yawen Bo, Hua Xu, Guiyuan Jiang, Kebin Chi

**Affiliations:** 1 CNPC Petrochemical Research Institute Company Limited, Beijing 102206, Chinalimengchen@petrochina.com.cn (M.L.);; 2 College of Chemical Engineering and Environment, China University of Petroleum-Beijing, Beijing 102249, China

**Keywords:** metal–organic frameworks, ultra-microporous, robust, breakthrough, CO_2_ capture

## Abstract

Developing metal–organic framework (MOF) adsorbents with excellent performance and robust stability is of critical importance to reduce CO_2_ emissions yet challenging. Herein, a robust ultra-microporous MOF, Cu(bpfb)(bdc), with mixed ligands of N, N′-(1,4-phenylene)diisonicotinamide (bpfb), and 1,4-dicarboxybenzene (bdc) was delicately constructed. Structurally, this material possesses double-interpenetrated frameworks formed by two staggered, independent frameworks, resulting in two types of narrow ultra-micropores of 3.4 × 5.0 and 4.2 × 12.8 Å^2^, respectively. The above structural properties make its highly selective separation at 273~298 K with a CO_2_ capacity of 71.0~86.2 mg/g. Its adsorption heat over CO_2_ and IAST selectivity were calculated to be 27 kJ/mol and 52.2, respectively. Remarkably, cyclic breakthrough experiments corroborate its impressive performance in CO_2_/N_2_ separation in not only dry but also 75% RH humid conditions. Molecular simulation reveals that C-H···O_CO2_ in the pores plays a pivotal role in the high selectivity of CO_2_ adsorption. These results point out the huge potential application of this material for CO_2_/N_2_ separation.

## 1. Introduction

With worldwide rapid industrialization, CO_2_ has become a major greenhouse gas and causes many serious problems, such as global warming and climate change, threatening the sustainable development of human society [1,2]. One of the most important reasons for incremental CO_2_ emission into the atmosphere is the burning of fossil fuels [3]. In particular, flue gas, which consists of ~15% CO_2_, 75% N_2_, and other impurities, released from power plants, steel, cement, and petrochemical industries, is a main source of CO_2_ emissions [4]. Hence, CO_2_ capture from flue gas is of great significance and urgency to achieve net zero emission goals, attracting considerable attention from scientists.

Among all separation technologies, absorption of CO_2_ by amines is the most popular way [5,6]. Although effective, it endures high energy costs during the regeneration process and causes pipeline corrosion. On the other hand, adsorption has milder performing conditions and lower energy costs, as well as simple equipment, providing an alternative technique [7]. As the highly close physical properties and kinetic diameters among CO_2_ (3.3 Å), N_2_ (3.65 Å), and other gas molecules, adsorbents possessing highly selective discrimination for CO_2_, as well as excellent stability from humidity, serve as the core in such a process. 

Metal–organic frameworks (MOFs) are promising adsorbents for gas separation, for their many attractive properties including high specific surface area and facile regulation of pore size and structure [8,9,10]. To date, a huge number of MOF adsorbents have been reported. Generally, introducing open metal sites (OMS, metal sites with solvent molecules coordinated or unsaturated) and grafting Lewis basic sites (-NH_2_, alkylamine, arylamine, etc.) or other functional polar sites (-F, -CN, -PF_6_, etc.), which could enhance MOF–CO_2_ interaction, are effective strategies to achieve high adsorption capacity or separation selectivity, but usually result in higher binding energy or adsorption heat (*Q*_st_) [11]. For instance, Mg-MOF-74 [12,13], with abundant OMS, reached a dramatic capacity of 8.0 mmol/g at 1 bar, 296 K. However, this MOF suffers from high *Q*_st_ value and vulnerability to moisture as a side-effect of the introduced OMS. Besides the above-mentioned strategies, molecular sieving through crystal engineering strategies on MOF, particularly for pore engineering by tuning the pore size, volume, shape, and surface, provides as an alternative way with a low *Q*_st_ value [14,15,16,17,18,19,20,21,22]. Generally, a MOF with a confined pore size (<4 Å) usually exhibits high selectivity but low adsorption capacity, while an expanded pore size (>4 Å) usually shows high adsorption capacity but low selectivity [19]. Therefore, in contrast to MOFs representing uniform 1D pore/channel, it might achieve an ideal balance between adsorption uptake and selectivity by constructing a MOF featuring two types of pores in size. However, precise control of a MOF’s pore/channel in the high resolution of 0.1 Å remains a great challenge. 

Based on these concepts, we synthesized an ultra-microporous pillar-layered MOF, Cu(bpfb)(bdc), termed PRI-1 (PRI stands for Petrochemical Research Institute), with dual pore sizes for efficient CO_2_/N_2_ separation. Firstly, the material’s crystal purity and porosity were characterized by PXRD (powder X-ray diffraction) and low-temperature CO_2_ adsorption isotherm. Secondly, the adsorbent’s thermal and chemical stability was investigated via TG and solvent-immersing tests. Thirdly, its adsorption isotherms for CO_2_ and N_2_ were characterized under various temperatures and the IAST model [23,24] was utilized to estimate its selectivity over CO_2_/N_2_ binary mixture at 298 K. Fourthly, its dynamic adsorption/separation performance for CO_2_/N_2_ mixtures in dry and highly humid conditions was studied. Finally, the molecular simulation was performed to reveal the adsorption mechanism of CO_2_/N_2_ on PRI-1.

## 2. Results and Discussion

### 2.1. Sample Characterization

**Figure 1:**
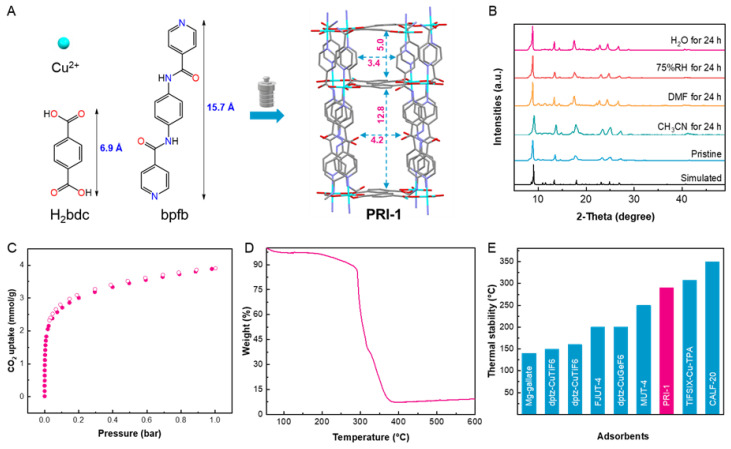
(**A**) The synthesis of PRI-1. (**B**) PXRD patterns of the simulated and as-synthesized PRI-1, as well as those after various treatments. (**C**) 195 K CO_2_ adsorption-desorption isotherms. (**D**) TGA curve. (**E**) Comparison of thermal stabilities of PRI-1 and other MOF adsorbents. The distances are in Å.

PRI-1 was facilely synthesized via solvothermal reaction from Cu(NO_3_)_2_·3H_2_O, bpfb, and H_2_bdc in DMF (Figure 1A and Figure S1). The PXRD patterns of as-synthesized PRI-1 and simulated (CCDC No: 641709) [25] were nearly identical (Figure 1B). The characteristic peaks of 9.0° and 13.3° of the as-synthesized PRI-1 agree well with the calculated peaks. Togethering with IR spectroscopy [25] (Figure S2), the PXRD patterns indicate the successful synthesis of PRI-1. Meanwhile, the mother liquor circulation synthesis method was successfully utilized to further reduce the synthesis cost of this material (Figure S3). The morphology and size of PRI-1 were revealed by scanning electron microscopy. Figure S4 shows that PRI-1 crystalized in rods with particle sizes in micrometers.

Structurally, this MOF is described by a *pcu*-type framework with double-interpenetrated nets. In each net, two Cu^2+^ cations serve as the metal center and are coordinated by four bdc bidentate ligands, forming the layer. In the axial orientation, four bpfb ligands are coordinated, serving as the pillar. In the occurrence to the twofold interpenetration, there are 3675.6 Å^3^ accessible voids (30.7% of the cell volume), as calculated using the Mercury software [26]. Meanwhile, PRI-1 possesses two types of 1D channels in parallel and their sizes are 3.4 × 5.0 and 4.2 × 12.8 Å^2^, respectively. These parameters are very close to the dynamic radius of CO_2_.

The CO_2_ adsorption-desorption isotherm of PRI-1 at 195 K showed the ultra-microporous structure of the material (Figure 1C) with adsorption reaching 87.5 cm^3^/g at 1 bar and a calculated BET surface area of 317.3 m^2^/g. The porosity endows the material with the ability to separate CO_2_ efficiently. 

The thermal stability of PRI-1 was examined via thermogravimetric analysis in an air atmosphere. As shown in Figure 1D, there is a minor weight loss starting from 30 to 290 °C, which is mainly attributed to the removal of guest solvents, and then a major weight loss emerged after 290 °C, indicating the framework collapse of the material. The TGA curve shows the high thermal robustness of the material until 290 °C, which is superior to most recently-reported CO_2_ adsorbents, including Mg-gallate (140 °C) [27], dptz-CuTiF6 (150 °C) [21], SIFSIX-3-Zn (160 °C) [28], FJUT-4 (200 °C) [29], and dptz-CuGeF_6_ (200 °C) [30], MUT-4 (250 °C) [31], as well as slight lower than that of TiFSIX-Cu-TPA (308 °C) [19] and CALF-20 (350 °C) [32] (Figure 1D).

### 2.2. CO_2_ and N_2_ Isotherms, Adsorption Heat, and Selectivity of PRI-1

**Figure 2:**
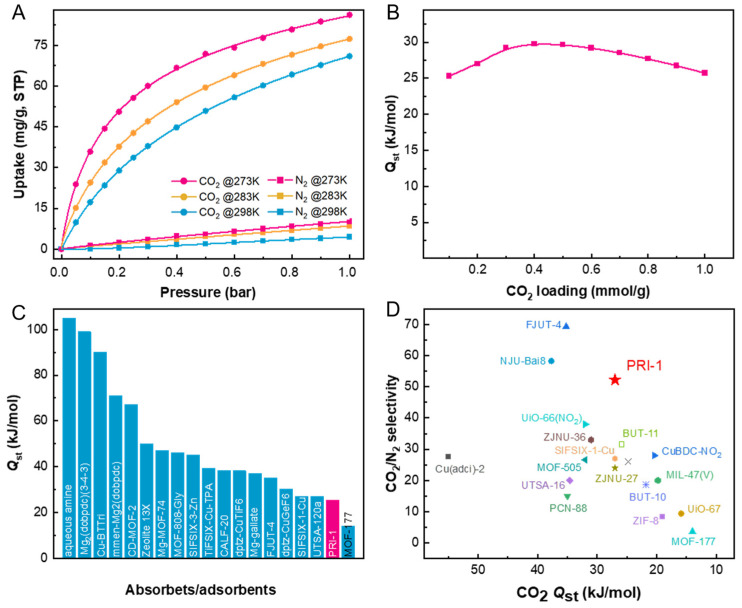
(**A**) CO_2_ and N_2_ adsorption isotherms of PRI-1 at 273, 283, and 298 K. (**B**) *Q*_st_ of CO_2_ in PRI-1. (**C**) Comparison of CO_2_ *Q*_st_ for PRI-1 with those of other top-performing MOF adsorbents. (**D**) Comparison of CO_2_/N_2_ (15:85, *v*/*v*) IAST selectivity at 298 K and 1 bar and *Q*_st_ of CO_2_ under ambient conditions in PRI-1 with those of reported CO_2_-selective MOFs.

Inspired by its structural features, the CO_2_ and N_2_ isotherms of PRI-1 were collected at 273, 283, and 298 K, respectively, as depicted in Figure 2A. The CO_2_ capacity was up to 71.0~86.2 mg/g at 1 bar, while only 8.1~10.2 mg/g for N_2_, suggesting an obviously preferential CO_2_ adsorption. The isosteric heat (*Q*_st_) of CO_2_ was calculated using the Clausius–Clapeyron equation [25] based on adsorption isotherms at 283 and 298 K, demonstrating a value of 27.0 kJ mol^−1^ at CO_2_ loading of 0.1 mmol/g (Figure 2B). Its *Q*_st_ value is much lower than aqueous amine (105 kJ/mol) [21] and many MOF adsorbents (Figure 2C), including Mg_2_(dobpdc)(3-4-3) (99 kJ/mol) [20], Cu-BTTri (90 kJ/mol) [33], mmen-Mg_2_(dobpdc) (71 kJ/mol) [34], CD-MOF-2 (67.2 kJ/mol) [35], MOF-808-Gly (46 kJ/mol) [36], Mg-MOF-74 (47 kJ/mol) [37], Zeolite 13X (44~54 kJ/mol), SIFSIX-3-Zn (45 kJ/mol) [28], FJUT-3 (41.7 kJ/mol) [38], TiFSIX-Cu-TPA (39.2 kJ/mol) [19], CALF-20 (38.4 kJ/mol) [32], dptz-CuTiF_6_ (38.2 kJ/mol) [21], Mg–gallate (37 kJ/mol) [27], FJUT-4 (35.2 kJ/mol) [29], dptz-CuGeF_6_ (30.3 kJ/mol) [30], etc. This low *Q*_st_ value would largely reduce the regeneration energy cost.

Based on the isotherm data, the ideal adsorbed solution theory (IAST) [23,24] was employed to qualitatively estimate the CO_2_/N_2_ selectivity of PRI-1. The dual-site Langmuir and Freundlich (DSLF) model was applied to fit the single component isotherms of CO_2_ and N_2_ at 298 K. Figures S5 and S6 show the fitting curves and parameters of the DSLF model. Figure S7 and Figure 2D show that the CO_2_/N_2_ selectivity of PRI-1 reached 52.2 (the detailed calculation procedures of IAST selectivity see Supplementary Excel file), higher than many MOFs in the literature.

### 2.3. Adsorption Mechanism of CO_2_ and N_2_ on PRI-1

**Figure 3:**
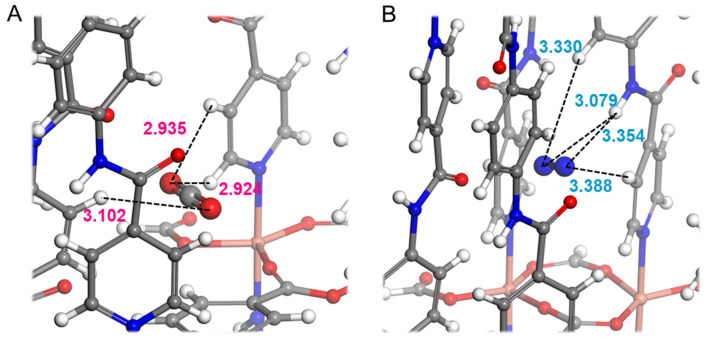
Calculated preferential binding sites for CO_2_ (**A**) and N_2_ (**B**) on PRI-1. The distances are in Å. Cu, C, N, O, and H atoms are shown in pink, grey, blue, red, and white, respectively.

DFT calculations were employed to elucidate the adsorption mechanism of carbon dioxide and nitrogen on PRI-1. Figure 3 illustrates the adsorption sites of CO_2_ and N_2_ on the PRI-1 framework. The figure reveals that CO_2_ molecules are captured within the main channels of PRI-1, showcasing the van der Waals interaction between gas molecules and hydrogen on the benzene ring and pyridine ring. The distance between the O···H-C bond is calculated as 2.924~3.102 Å, as depicted in Figure 3A, thereby highlighting the robust gas–ligand interactions during the adsorption process. This interaction arises from multi-directional hydrogen bond donors, which collectively confine the adsorption of CO_2_ molecules within the pores of PRI-1. Simultaneously, N_2_ molecules are captured within the main channels of PRI-1 due to the influence of hydrogen originating from the same side pyridine ring and amide group, exhibiting an N...H bond distance of 3.079~3.388 Å, as depicted in Figure 3B. Compared to the multi-directional adsorption of CO_2_, the unidirectional adsorption of N_2_ is relatively weaker within the PRI-1 framework. Furthermore, the calculated adsorption energies of carbon dioxide and nitrogen on PRI-1 amount to 28.44 kJ/mol and 15.52 kJ/mol, respectively, aligning with the experimental findings. PRI-1 possesses a distinctive pore structure and multiple ligands that facilitate extensive interactions between gas molecules and the framework, rendering it an excellent MOF material for the efficient separation of CO_2_/N_2_. 

### 2.4. Breakthrough Curve of CO_2_/N_2_ Binary Mixtures

**Figure 4:**
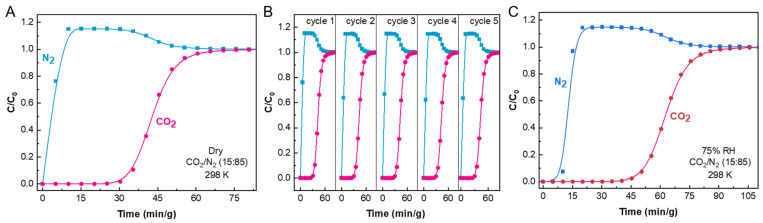
(**A**) Experimental breakthrough curves of PRI-1 for CO_2_/N_2_ (15:85) binary mixture at 298 K and 1 bar. (**B**) Experimental cycling breakthrough curves of a CO_2_/N_2_ (15:85) binary mixture at 298 K and 1 bar. (**C**) Experimental breakthrough for CO_2_/N_2_ (15:85) under both dry and 75% RH humid conditions. C and C_0_ stand for outlet concentration and inlet concentration of CO_2_ and N_2_ in the gas mixture flow, respectively.

To evaluate the dynamic separation properties of PRI-1, breakthrough tests were carried out with a simulated binary mixture of CO_2_/N_2_ (15:85) at 298 K. Figure 4 presents the breakthrough curve at 298 K of the binary mixture through a fixed bed of PRI-1. It was obvious that N_2_ was eluted out almost immediately after the gas mixture fed in, while CO_2_ was retained for 30.3 min/g (Figure 4A) under the flow rate of 2 mL/min, superior to the separation performance to many MOFs including the recently reported molecular sieving adsorbents, FJUT-4 [23]. As recyclability is of great importance for practical use, the breakthrough experiments were repeated for five cycles and the separation performance displayed a negligible loss (Figure 4B). 

More importantly, the separation properties in humid conditions also play a vital role in its practical use. Considering its robustness in various harsh solvents as mentioned above, we further conducted the breakthrough experiments under humid conditions. Similar to the robust ZIF-94 [39] and FJUT-3 [38], the presence of humidity (75% RH, 298 K) did not significantly alter the CO_2_ breakthrough time for PRI-1 (Figure 4C), showing efficient CO_2_/N_2_ separation ability in both dry and humid conditions. 

## 3. Conclusions

In this paper, an ultra-microporous MOF, PRI-1, was facilely synthesized via a solvothermal method for efficient separation of CO_2_/N_2_. Structurally, PRI-1 possesses double-interpenetrated frameworks formed by two staggered nets, resulting in dual pore sizes. PRI-1 shows its high thermal stability up to 290 °C, as well as its structural robustness from moist air and various polar solvents, including water. Its CO_2_ and N_2_ uptake were 71.0~86.2 mg/g and 8.1~10.2 mg/g at 1 bar and 273~298 K, respectively. *Q*_st_ of CO_2_ was calculated to be 27.0 kJ/mol at the loading of 0.1 mmol/g. The calculated IAST selectivity for CO_2_/N_2_ (15:85, *v*/*v*) was 52.2 at 298 K and 1 bar. The breakthrough experiments prove it possesses excellent dynamic separation properties under both dry and 75% RH humid conditions. Molecular simulation revealed that the tight C-H…O_CO2_ interaction contributes. This work proves that the exquisite construction of an ultra-microporous MOF with dual pore sizes might be an efficient and facile strategy for efficient CO_2_ separation.

## 4. Materials and Methods

### 4.1. Materials

Copper (II) nitrate hexahydrate (Cu(NO_3_)_2_ 3H_2_O, 99%), N,N-Dimethylformamide (DMF, 99.5%), and methanol (CH_3_OH, 99%) were purchased from Sinopharm Chemical Reagent Co., Ltd. (Shanghai, China). Benzene-1,4-dicarboxylic acid (H_2_bdc, 99%) was purchased from Shanghai Aladdin Biochemical Technology Co., Ltd. (Shanghai, China). N,N′-(1,4-phenylene)diisonicotinamide (bpfb, 95%) was purchased from Jilin Chinese Academy of Sciences—Yanshen Technology Co., Ltd. (Changchun, China). All reagents were used directly without any purification.

### 4.2. Synthesis of PRI-1

The synthesis procedure of PRI-1 was optimized from previous literature [25]. Firstly, Cu(NO_3_)_2_ 3H_2_O (0.5 mmol, 0.121 g), bpfb (0.5 mmol, 0.159 g), and H_2_bdc (0.5 mmol, 0.083 g) were dissolved in DMF (20 mL) and under ultrasonicated for 30 min at room temperature. Afterward, the resulting brown clear solution was transferred into the autoclave and heated at 393 K for 48 h, and then cooled down at the rate of 3 K/h. The obtained brown powders were washed with DMF and CH_3_OH thrice, respectively. After being soaked in CH_3_OH for 1 day and heated at 373 K under vacuum for 12 h, 0.259 g of sample was obtained at a yield of 94.9% (based on Cu).

### 4.3. Mother Liquor Circulation Synthesized Method

The mother liquor after the first reaction was reused for the next turn of PRI-1 synthesis. Cu(NO_3_)_2_ 3H_2_O (0.25 mmol, 0.061 g), bpfb (0.25 mmol, 0.080 g) and H_2_bdc (0.25 mmol, 0.042 g) were dissolved into the filtered mother liquor and ultrasonicated for 30 min at room temperature. Afterward, the resulting brown clear solution was transferred into the autoclave and heated at 393 K for 48 h, as above. The sample was obtained with a yield of 78.4%. The mother liquor circulation synthesized method would significantly reduce the cost of organic solvents in synthesis.

### 4.4. Characterization

The crystal structure and crystallinity of the sample were measured using PXRD on an X’Pert PRO powder diffractometer (PANalytical, Almelo, The Netherlands) with a Cu Kα radiation source (λ = 1.5418 Å). Scanning was performed over a 2θ range of 5~50° at a scanning rate of 2°/min. Thermogravimetric analysis (TGA) of the sample was carried out on a thermal analyzer (HCT-4, Beijing Hengjiu Experimental Equipment Co., Ltd. Beijing, China). The sample was heated at a rate of 5 °C/min under an air atmosphere. The morphology of the as-synthesized sample was observed and confirmed using SEM on a JEOL JSM-IT200 instrument. CO_2_ adsorption–desorption isotherms were recorded at 195 K on BSD-PM instruments. Before measurement, the sample was degassed at 100 °C for 12 h and all gases were of 99.999% purity.

### 4.5. Adsorption Isotherms Measurement

Single-component adsorption isotherms of CO_2_ and N_2_ were collected on a vacuum vapor/gas sorption analyzer (BSD-VVS, 3H-2000PW). Before each measurement, the sample was degassed and activated at 373 K under vacuum for at least 6 h until no weight loss was observed and subsequently was cooled to room temperature. High purity (99.9999%) CO_2_ and N_2_ were utilized in the gas adsorption experiments.

### 4.6. Solvent Stability Test

The stability of the samples was investigated by PXRD after being immersed into acetonitrile, DMF, methanol, and DI water for 24 h, respectively.

### 4.7. Fixed-Bed Breakthrough Experiments

The breakthrough curves of the gas mixture CO_2_/N_2_ (15:85, *v*/*v*) were tested on homemade dynamic breakthrough equipment (Figure S8). Similar to the packing technique in recent articles [19,27], 460 mg of dry sample was filled in a stainless-steel HPLC adsorption column (Φ4.6 × 50 mm). Before the experiment, the column was purged and activated under flowing He gas at 373 K overnight. During the experiment, the above gas mixture was injected with a flow rate of 2 mL min^−1^. The out-gas mixture was monitored in real time by a gas chromatography apparatus (7890A, Agilent Technologies, Inc. Santa Clara, CA, USA), equipped with a HayeSep Q column and a TCD detector. After each test, the column was regenerated with He gas flow at room temperature for 30 min, and the cycling breakthrough tests were performed under identical conditions as above.

### 4.8. Fitting with Dual-Site Langmuir-Freundlich Model

Single-component adsorption isotherms of CO_2_ and N_2_ on PRI-1 obtained at different temperatures were fitted using the dual-site Langmuir-Freundlich model [40] through Origin software:

(1) $$n=\frac{N_{1}\times a\times p^{b}}{1+a\times p^{b}}+\frac{N_{2}\times c\times p^{d}}{1+c\times p^{d}}$$

where *n* is the equilibrium amount adsorbed in mmol/g, *p* is the equilibrium pressure in kPa, *N*_1_, and *N*_2_ is the adsorbed amount at site 1 and site 2, respectively; *a* is the maximal loading in mmol/g, *a* and *c* are the affinity constants of site 1 and site 2, respectively; and 1/*b* and 1/*d* are the deviations from an ideal homogeneous surface.

### 4.9. Ideal Adsorbed Solution Theory Calculations

In the context of IAST, it is postulated that the adsorbed mixture behaves as an ideal solution under constant spreading pressure and temperature. According to this theory, all components within the mixture conform to a rule similar to Raoult’s law, and the chemical potential of the adsorbed solution is assumed to be in equilibrium with that of the gas phase.

From IAST, the spreading pressure *π* is given by

(2) $$\pi_{i}^{0}\left(p_{i}^{0}\right)=\frac{RT}{A}\int_{0}^{p_{i}^{0}}ad\ln p$$

(3) $$\pi^{*}=\frac{\pi A}{RT}=\int_{0}^{p_{i}^{0}}\frac{q_{i}}{p}dp$$

where *A* is the specific surface area of the adsorbent, *π* and π* are the spreading pressure and reduced spreading pressure, respectively. $p_{i}^{0}$ represents the individual gas pressures corresponding to component *i* at a given spreading pressure *π* of the gas mixture. 

Under constant temperature, the spreading pressure remains constant for each individual component.

(4) $$\pi_{1}^{*}=\pi_{1}^{*}=\cdots =\pi_{n}^{*}=\pi$$

In the case of binary adsorption involving component 1 and component 2, the IAST formulation requires the following expression,

(5) $$y_{1}p_{t}=x_{1}p_{1}\;(1-y_{1})p_{t}=\left(1-x_{1}\right)p_{2}$$

where *y*_1_ and *x*_1_ represent the molar fractions of component 1 in the gas phase and adsorbed phase, respectively. The total gas pressure is denoted as *p*_t_, while *p*_1_ and *p*_2_ represent the pressures of component 1 and component 2 at the same spreading pressure as that of the mixture.

The adsorption selectivity in a binary mixture of component 1 and component 2 can be defined as

(6) $$S_{ads}=\frac{x_{1}/x_{2}}{y_{1}/y_{2}}$$

where *x*_1_ and *x*_2_ represent the component molar loadings within the MOF, and *y*_1_ and *y*_2_ are the corresponding mole fraction in the bulk phase, respectively.

### 4.10. Calculation of Isosteric Heat of Adsorption

The isosteric heat of adsorption was calculated by analyzing the fitted adsorption isotherms at three different temperatures (i.e., 273 K, 283 K, 298 K) based on the Clausius–Clapeyron equation. The equation can be presented as follows:

(7) $$lnp=-\frac{∆H_{S}}{RT}+C$$

where *p* is the pressure (kPa), $∆H_{S}$ is the isosteric heat of adsorption at a given loading (kJ/mol), *R* is the ideal gas constant (8.314 kJ/mol/K), *T* is the temperature (K) and *C* is the integral constant.

### 4.11. Computational Method 

All DFT calculations in this work utilize the VASP package [41,42] with the generalized gradient approximation (GGA) based on the Perdew–Burke–Ernzerhof (PBE) functional [43]. Valence electrons were simulated utilizing projector-augmented-wave (PAW) pseudopotentials [42,44], with a cutoff energy of 400 eV. A 3 × 3 × 2 Monkhorst–Pack *k*-point mesh sampling was utilized during simulations [45]. The required structural optimization accuracy was reached when the forces on the relaxed atoms were less than 0.05 eV/Å. The DFT-D3 approximation method was utilized to correct van der Waals (vdW) interactions [46]. Adsorption energies are calculated as Δ*E*_ads_ = *E_MOF_*_+gas_ − (*E_MOF_* + *E*_gas_), where *E*_*MOF*+gas_ is the total energy of the adsorption complex, and gas represents the CO_2_ or N_2_. 

## Data Availability

The data presented in this study are available on request from the corresponding author.

## Supplementary Materials

The following supporting information can be downloaded at: https://www.mdpi.com/article/10.3390/molecules28176276/s1, Figure S1. The asymmetric unit of PRI-1. Figure S2. The FT-IR spectra of Cu(bpfb)(bdc) and raw materials. Figure S3. PXRD patterns of PRI-1 samples from the fresh synthesis and the mother liquor circulation synthesized method. Figure S4. SEM pictures of PRI. Figure S5. Gas adsorption isotherm of CO_2_ with the DSLF fit for PRI-1 at 273, 283 and 298 K. Figure S6. Gas adsorption isotherm of N_2_ with the DSLF fit for PRI-1 at 273, 283 and 298 K. Figure S7. IAST selectivity of PRI-1 for CO_2_/N_2_ (15:85) at 298 K. Figure S8. Diagram of the homemade dynamic breakthrough experimental apparatus. Supplementary Excel file. Detailed calculation procedures of IAST selectivity.

## Sample Availability

Samples of the compounds are not available from the authors.
