# Supplementary material for: Exquisitely Constructing a Robust MOF with Dual Pore Sizes for Efficient CO2 Capture
Source: Molecules. 2023 Aug 28;28(17):6276. doi: 10.3390/molecules28176276 (PMC10488667; doi:10.3390/molecules28176276)
Supplement: Supplementary file 1 [file molecules-28-06276-s001.zip › molecules-2551875-supplementary.pdf]

Exquisitely Constructing a Robust MOF with Dual Pore Sizes for Efficient CO_2 Capture

Yanxi Li [1,*], Yuhua Bai [1,2], Zhuozheng Wang [1], Qihan Gong [1,*], Mengchen Li [1], Yawen Bo [1], Hua Xu [1], Guiyuan Jiang [2] and Kebin Chi [1,*]

[1] CNPC Petrochemical Research Institute Company Limited, Beijing 102206, China; limengchen@petrochina.com.cn (M.L.)
[2] College of Chemical Engineering and Environment, China University of Petroleum-Beijing, Beijing 102249, China
* Correspondence: liyanxi@petrochina.com.cn (Y.L.); gongqihan@petrochina.com.cn (Q.G.); ckb459@petrochina.com.cn (K.C.)

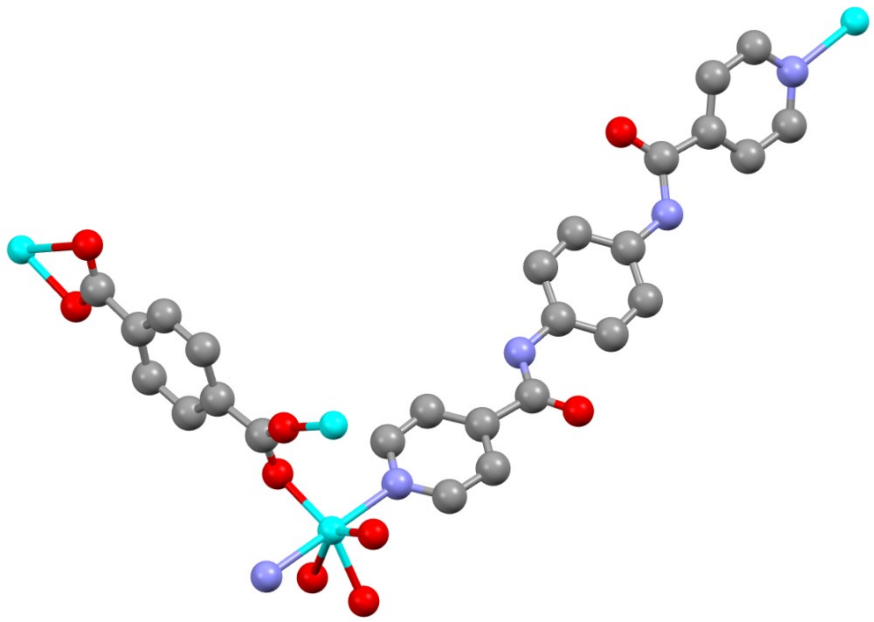

Figure S1. The asymmetric unit of PRI-1. Cu, C, N, O atoms are in cyan, grey, blue, and red, respectively.

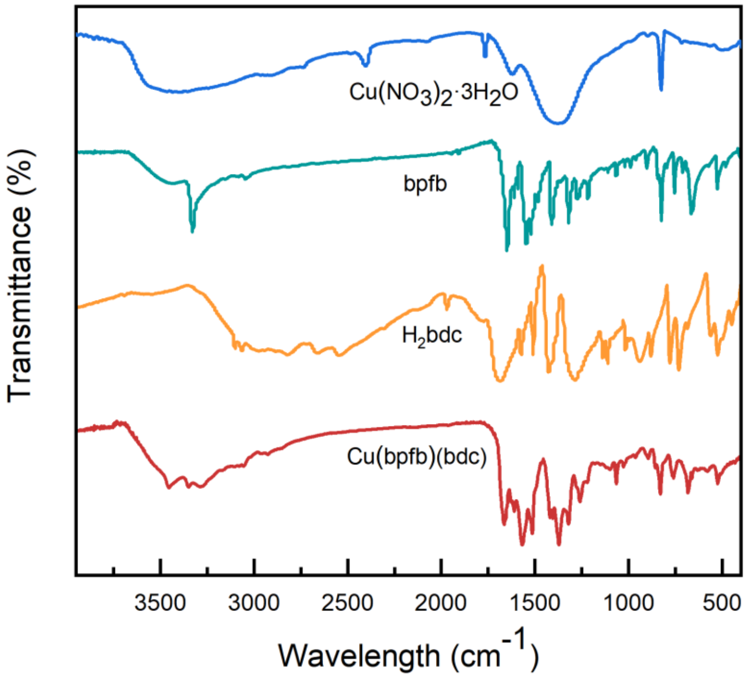

Figure S2. The FT-IR spectra of PRI-1 and raw materials.

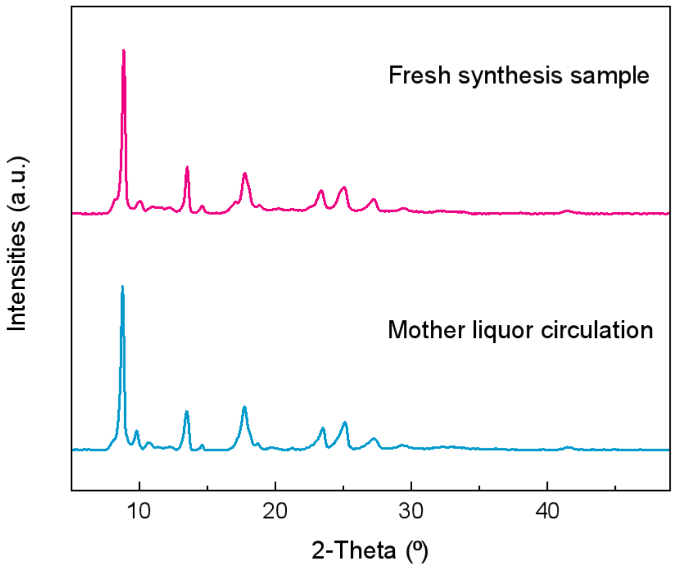

Figure S3. PXRD patterns of PRI-1 samples from the fresh synthesis and Mother liquor circulation synthesized method.

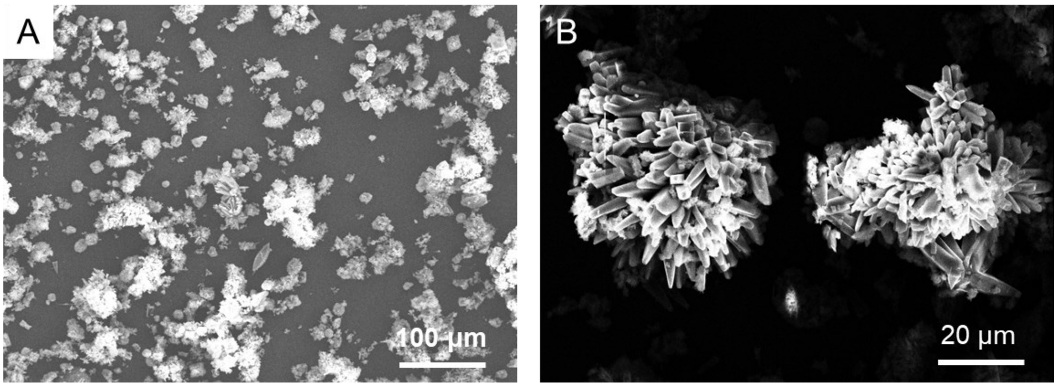

Figure S4. SEM pictures of PRI.

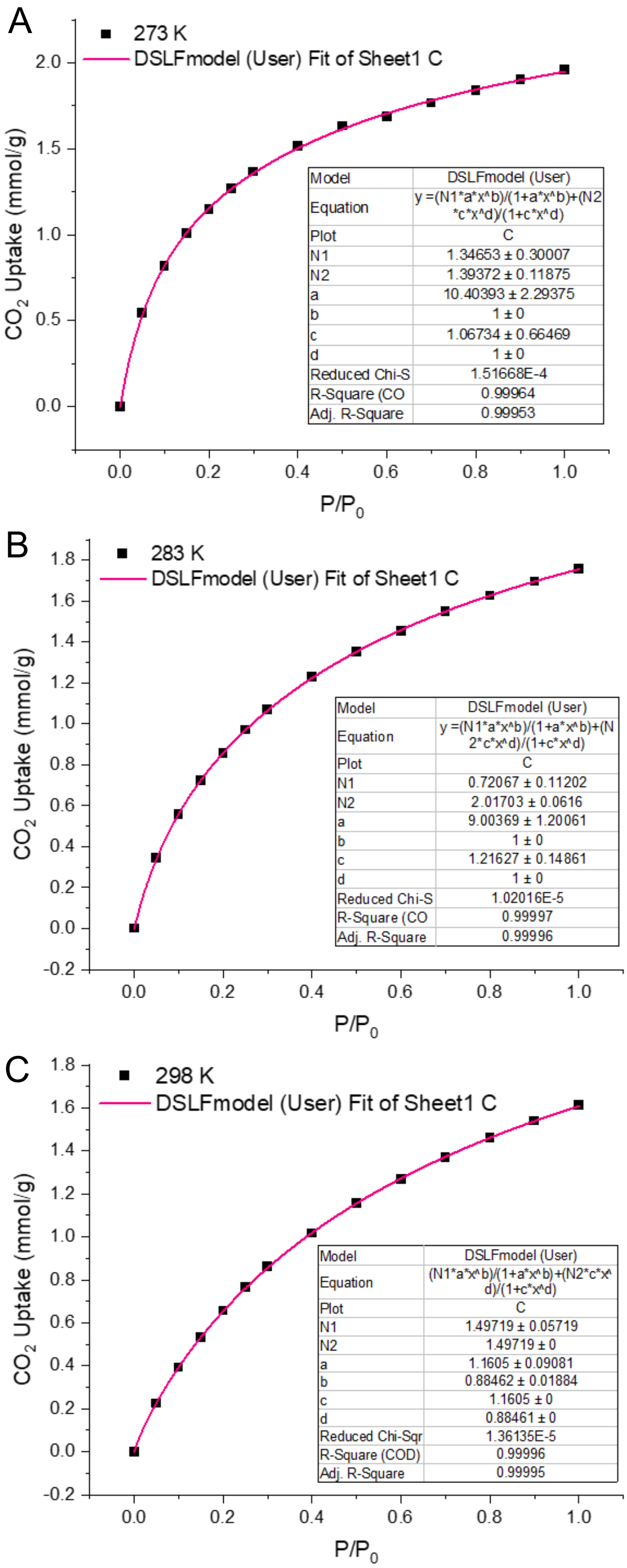

Figure S5. Gas adsorption isotherm of CO_2 with the DSLF fit for PRI-1 at 273, 283 and 298 K.

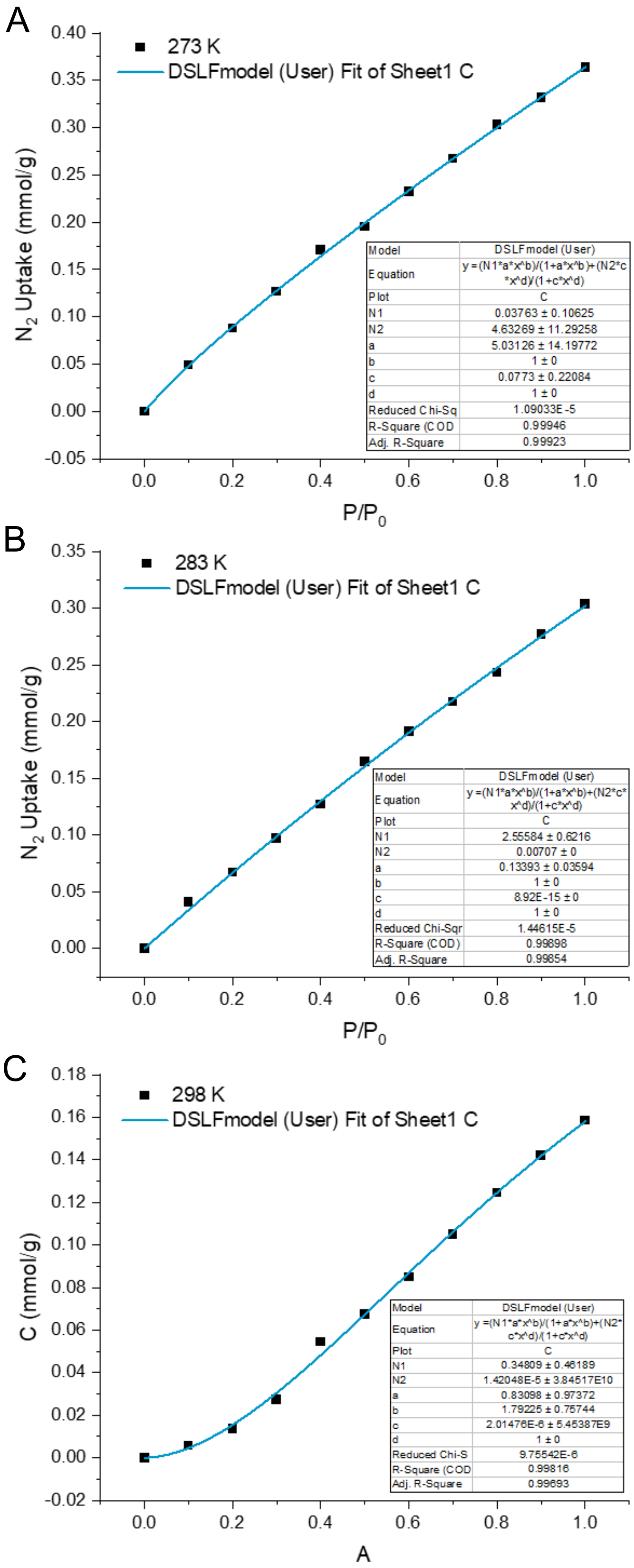

Figure S6. Gas adsorption isotherm of N_2 with the DSLF fit for PRI-1 at 273, 283 and 298 K.

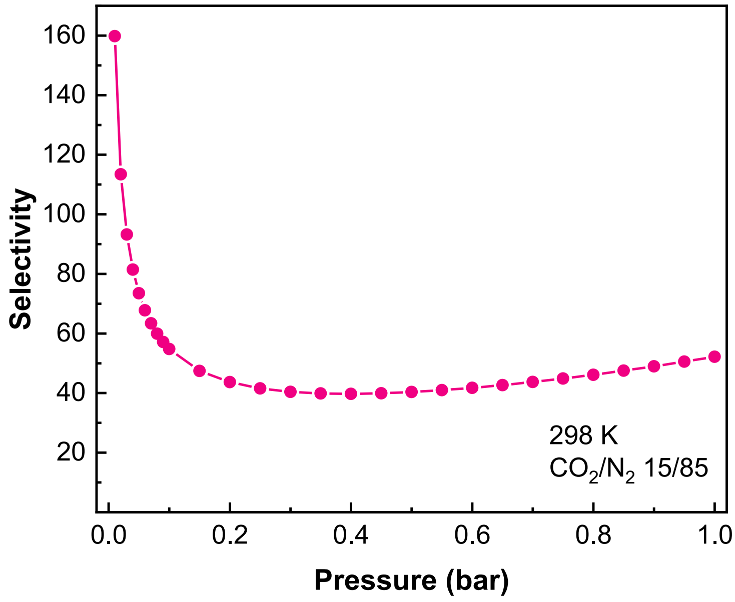

Figure S7. IAST selectivity of PRI-1 for CO_2/N_2 (15:85) at 298 K.

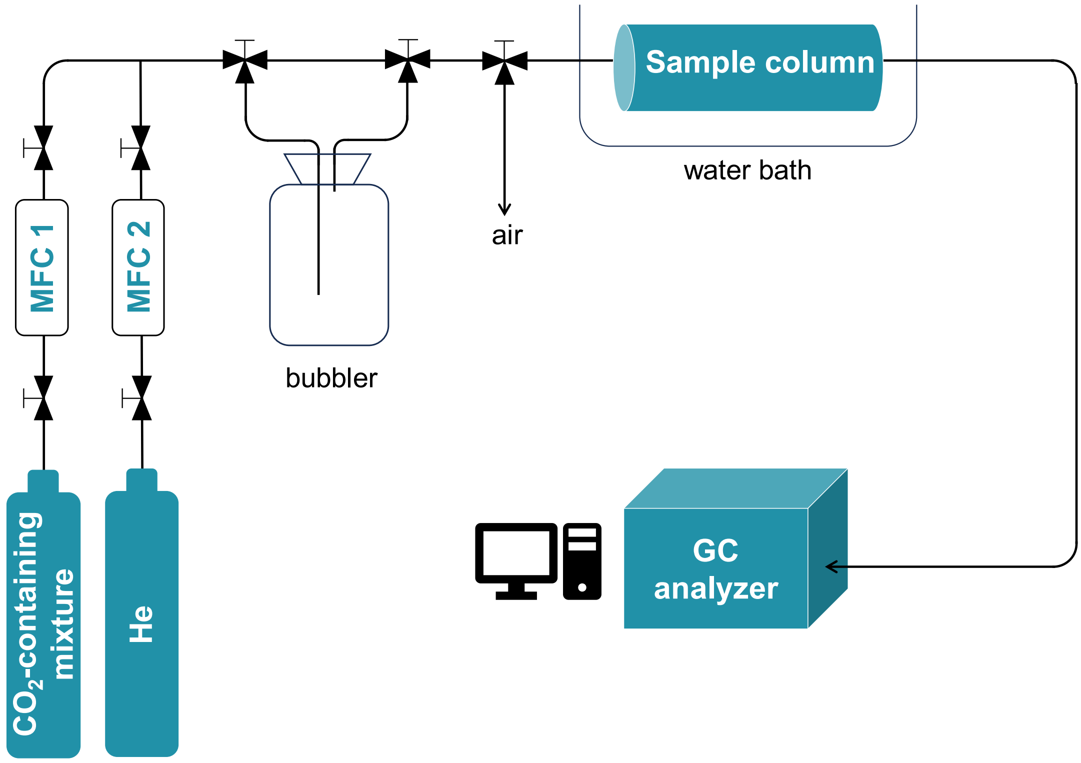

Figure S8. Diagram of the home-made dynamic breakthrough experimental apparatus.
